# Antibacterial sensitizers from natural plants: A powerful weapon against methicillin-resistant *Staphylococcus aureus*

**DOI:** 10.3389/fphar.2023.1118793

**Published:** 2023-02-24

**Authors:** Xiaoli Li, Yongqing Cai, Qinchuan Xia, Yongqun Liao, Rongxin Qin

**Affiliations:** ^1^ Department of Pharmacology, College of Pharmacy, Chongqing Medical University, Chongqing, China; ^2^ Chongqing Key Laboratory of Drug Metabolism, Chongqing, China; ^3^ Department of Pharmacy, Daping Hospital, Army Medical University, Chongqing, China; ^4^ Fuan Pharmaceutical Group Chongqing Bosen Pharmaceutical Co., Ltd., Chongqing, China; ^5^ Department of Pharmacology, College of Pharmacy, Army Medical University (The Third Military Medical University), Chongqing, China

**Keywords:** MRSA, antibacterial sensitizers, natural products, PBP2a, efflux pumps

## Abstract

Methicillin-resistant *Staphylococcus aureus* (MRSA) is a drug-resistant bacterium that can cause a range of infections with high morbidity and mortality, including pneumonia, *etc.* Therefore, development of new drugs or therapeutic strategies against MRSA is urgently needed. Increasing evidence has shown that combining antibiotics with “antibacterial sensitizers” which itself has no effect on MRSA, is highly effective against MRSA. Many studies showed the development of antibacterial sensitizers from natural plants may be a promising strategy against MRSA because of their low side effects, low toxicity and multi-acting target. In our paper, we first reviewed the resistance mechanisms of MRSA including “Resistance to Beta-Lactams”, “Resistance to Glycopeptide antibiotics”, “Resistance to Macrolides, Aminoglycosides, and Oxazolidinones” *etc.* Moreover, we summarized the possible targets for antibacterial sensitizers against MRSA. Furthermore, we reviewed the synergy effects of active monomeric compounds from natural plants combined with antibiotics against MRSA and their corresponding mechanisms over the last two decades. This review provides a novel approach to overcome antibiotic resistance in MRSA.

## 1 Introduction

*Staphylococcus aureus* is one of the most infectious Gram-positive bacteria affecting people in hospitals and communities (de Oliveira Santos et al., 2022). Owing to its relatively high virulence and plasticity, *S. aureus* can adapt to various environmental conditions, and is resistant to almost all antimicrobial drugs (Mlynarczyk-Bonikowska et al., 2022).

The emergence of methicillin-resistant *Staphylococcus aureus* (MRSA) strains makes treatment of infections more complicated. MRSA can cause a series of infections such as bacteraemia, pneumonia, as well as complicated skin and soft-tissue infections, which are difficult to treat resulting in high morbidity and mortality (Liang et al., 2022). Furthermore, treatment failure results in enormous medical costs. Therefore, there is an urgent need to develop new and more effective antibacterial strategies to eradicate MRSA.

At present, there are two main antibacterial strategies. The first is “direct antibacterial strategy”, that is, structural modification of existing antimicrobials to obtain compounds with higher activity and fewer side effects. Using this strategy, oxazolidinones, glycopeptides, quinolones, aminoglycosides, tetracyclines, and ketonolactone analogues have entered the clinic (Theuretzbacher, 2011). Unfortunately, with the promotion of their clinical use, there has been a gradual emergence of resistance owing to the selection pressure of antibiotics or the spread of bacteria. For example, simultaneous resistance to vancomycin, daptomycin, and ceftaroline was recently identified in MRSA (Wüthrich et al., 2019). Therefore, it is risky to develop antimicrobials with new targets and skeletons (Warren, 2011). The second antibacterial strategy is the “indirect antibacterial strategy”, which involves the development of drugs (antibacterial sensitizers) that do not themselves have antibacterial activity but can enhance the activity of existing antimicrobial agents. These drugs act by changing or modifying the phenotype of bacteria, thereby allowing existing antimicrobials to inhibit or kill them (Qin et al., 2013). Because antibacterial sensitizers do not exert direct selection pressure on bacteria, they do not induce the production of drug-resistant strains. Thus, antimicrobial sensitizers can enhance the antibacterial ability of existing antibiotics, restore their viability against drug-resistant strains, and reduce dependency on antibiotics (Tyers and Wright, 2019). The clinical application of antibacterial sensitizers has had a profound impact on the development of antimicrobial agents.

Plants are a huge treasure trove of antibacterial molecules and sensitizers against MRSA. Plants contain a wide variety of secondary metabolites that can resist external stress and pathogenic attacks. These compounds are classified into terpenoids, alkaloids, flavonoids, polyphenols, coumarins, and fatty acids (Shin et al., 2018). The advent of high-throughput screening methods for the assessment of large amounts of plant extracts containing putative biologically active compounds has encouraged industrial interest in plant research. Numerous studies have reported plant-derived small molecules that increase the sensitivity of MRSA to antibiotics. This could be a new strategy for overcoming MRSA infections.

This study reviewed the pharmacological action and preliminary mechanism of small molecular antibacterial sensitizers from natural products (excluding crude extract from plants) in the last 20 years. The aim of this review was to provide new ideas for the research and development of new antibacterial sensitizers.

## 2 Synergy and antagonism

Synergy is an important concept in the description of the effects of drug combinations and forms the basis for most antibiotic combinations. Synergy occurs when paired combinations of agents exert inhibitory effects that are more than the sum of their effects alone. Antagonism is the opposite of synergy, occurring when the combined activity of components is less than the sum of their individual activities. Synergy and antagonism are calculated using the fractional inhibitory concentration index (FICI) (Odds, 2003). A value of FICI≤0.5 is required for synergy and FICI ≥4.0 is required for antagonism (Tyers and Wright, 2019).

## 3 Resistant mechanisms of MRSA

### 3.1 Resistance to beta-lactams

Resistance to β-lactams is the most important mechanism of MRSA resistance. β-lactams targeted at penicillin-binding proteins (PBPs) which are responsible for peptidoglycan (PG) synthesis. PG is the essential component of the cell wall, providing the strength to resist high internal osmotic pressure and maintain cell shape in *S. aureus*. The inhibition of bacterial cell-wall biosynthesis by β-lactams will lead to bacterial cell death.

Several mechanisms of resistance are used, including the synthesis of β-lactamase and a new PBP (called PBP2a), and mutations in PBP genes (Mlynarczyk-Bonikowska et al., 2022).

#### 3.1.1 Synthesising the beta-lactamases

MRSA commonly synthesises specific hydrolases called β-lactamases, for resistance to β-lactams. The β-lactam ring can be hydrolysed by β-lactamases, which inactivates the β-lactams (Bush and Bradford, 2020). β-lactamases synthesised by *S. aureus* were classified as group 2a (Bush and Jacoby, 2010) and class A by Ambler (Tooke et al., 2019). At present, β-lactamase inhibitors have been successfully used in the clinic, including clavulanic acid, sulbactam, tazobactam, avibactam, and vaborbactam (Yahav et al., 2020), and many β-lactamase inhibitors are being investigated in clinical studies (Tooke et al., 2019).

#### 3.1.2 Expressing PBP2a and its Co-Factors

The most important factor mediating the resistance of MRSA to β-lactams is obtaining the *mecA* gene that can express penicillin-binding protein 2a (PBP2a). PBP2a has a very low affinity for β-lactams, but can compensate for the transpeptidase function of PBP2, which is inhibited by β-lactam antibiotics, to maintain the synthesis of the bacterial cell wall leading to bacterial resistance to almost all β-lactam drugs (Vestergaard et al., 2019). However, in several cases, differences in resistance levels did not correlate with the PBP2a expression levels, suggesting that factors other than PBP2a modulate strain-specific levels of β-lactam resistance (Luo et al., 2020; Zhang et al., 2020). Indeed, genetic screening has identified several auxiliary factors essential for methicillin resistance, which are also critical for PBP2a-mediated resistance to β-lactam antibiotics (Roemer et al., 2013). In general, these factors are divided into three categories: (A) factors related to *S. aureus* cell wall biogenesis, (B) factors related to *S. aureus* cell wall teichoic acid synthesis, and (C) other factors. Table 1 lists the factors affecting β-lactam resistance induced by PBP2a.

(A) Factors related to *S. aureus* cell wall biogenesis

**TABLE 1:** PBP2a relevant factors that can induce beta-lactam resistance.

Categories	Gene	Function
Factors related to cell wall biogenesis	*femX*	Peptidyltransferase, addition of first glycine to pentaglycine bridge
	*femA*	Peptidyltransferase, addition of 2nd and 3rd glycine to pentaglycine bridge
	*femB*	Peptidyltransferase, addition of 4th and 5th glycine to pentaglycine bridge
	*murA*	Transferase; converts UDP-GlcNAc to UDP-GlcNAc-enoylpyruvate
	*murB*	Reductase, converts UDP-GlcNAc-enoylpyruvate to UDP-MurNAc
	*murC-F*	Mur ligases adding L-Ala, D-Glu, L-Lys, and D-Ala-D-Ala respectively to form the UDP-MurNAc-pentapeptide
	*pbp1*	PBP with transpeptidation activity
	*pbp2*	PBP with transglycosylation and transpeptidation activity
	*pbp4*	PBP with transpeptidation activity
	*tarO*	Phosphosugar transferase; forms first precursor in WTA synthesis (GlcNAc-pyrophosphate-undecaprenol)
Factors related to the wall teichoic acid synthesis	*tarA*	N-acetylmannosamine transferase; forms second intermediate in WTA synthesis
	*tarB*	Glycerophosphate transferase; forms third intermediate in WTA synthesis
	*tarD*	WTA synthesis, produces CDP-glycerol substrate for TarB
	*tarL*	Ribitolphosphotransferase, required for poly ribitol-phosphate extension of WTA
Other factors	*ltaS*	Lipoteichoic acid synthase
	*vraSR*	two-component signal transduction systems
	*ftsZ*	the tubulin-like protein that forms the Z-ring involved in cell division
	*prsA*	the peptidyl-prolyl cis-trans isomerase to help PBP2a to fold correctly
	*floA*	role in membrane microdomain assembly
	*htrA1*	protease involved in PBP2a quality control
	*pknB*	Eukaryotic-like serine/threonine kinase
	*spsB*	Signal peptidase I
	*sigB*	Alternate transcription factor

**FIGURE 1:**
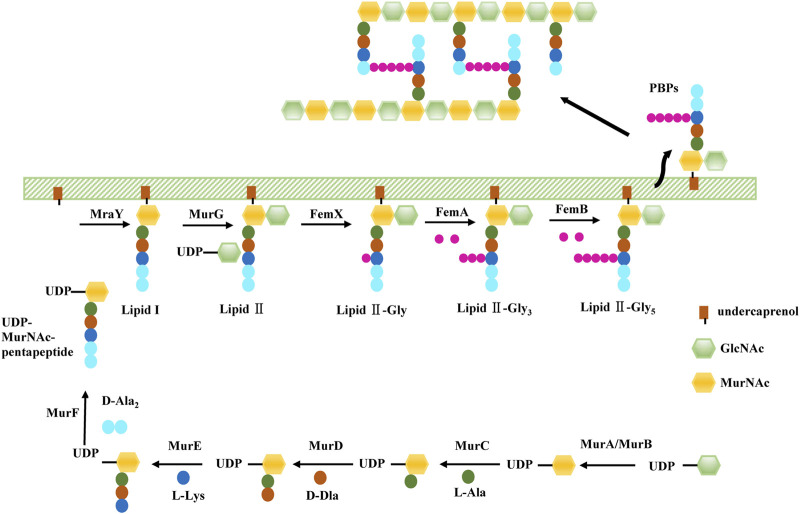
The biosynthesis of *S. aureus* cell wall.

As we mentioned before, PG, the target of β-lactams, maintain cell shape in *S. aureus.* The PG monomer is synthesised within the cytoplasm *via* a multistep process (Figure 1). First, UDP-GlcNAc is converted to UDP-MurNAc through the activities of MurA and MurB. PG ligases (MurC-F) then sequentially synthesise a pentapeptide (L-Ala-D-Glu-L-Lys-D-Ala-D-Ala), forming UDP-MurNAc-pentapeptide, the soluble PG precursor (van Heijenoort, 2001). Then, UDP-MurNAc-pentapeptide is linked to a lipid carrier by the membrane protein MraY, forming lipid I. Finally, MurG utilises the UDP-GlcNAc substrate to build the final PG precursor, lipid II (van Heijenoort, 2001; van Heijenoort, 2007). The *S. aureus* lipid II is further modified by a family of peptidyltransferases (FemX, FemA, and FemB) which completes a pentaglycine bridge peptide extending from the pentapeptide L-Lys residue, ultimately allowing for the efficient cross-linking of PG in the cell wall (Rohrer and Berger-Bächi, 2003). FemA installs the second and third glycines, forming Gly3-Lipid II, and FemB installs the fourth and fifth glycines, forming Gly5-Lipid II (Severin et al., 2005). Gly5-Lipid II is then flipped to the extracellular surface of the membrane, where it is polymerised and cross-linked catalysed by PBPs, including PBP2a in the case of MRSA. Because bacterial cell wall PGs are not found in mammalian cells, these inhibitors show highly selective toxicity to target cells.

In the process of *S. aureus* cell wall biogenesis, the peptidyl transferase family (FemX, FemA, FemB) and the Mur family (MurA, MurB, and MurC-F) are two important targets of antibacterial sensitizers.

(B) Factors related to *S. aureus* Wall Teichoic Acid synthesis

Wall teichoic acid (WTA) is a glycophosphate-rich cell-wall polymer common to Gram-positive bacteria (Swoboda et al., 2010). WTA polymers are sequentially synthesised on the central undecaprenyl phosphate carrier lipid C55-P by a series of Tar enzymes in the cytoplasm and then exported to the cell surface by a two-component ABC transporter system and cross-linked to PG by the LCP protein family (Pasquina et al., 2013). *S. aureus* WTA is not required for cell survival, but cells lacking WTA cannot complete cell division efficiently, lack fitness, reduce virulence, and become sensitive to β-lactams because PBP2a relies on glycosylated WTA to act as a scaffold/platform (Weidenmaier et al., 2004; Brown et al., 2012; Sewell and Brown, 2014). Thus, inhibitors of WTA synthesis act synergistically with beta-lactams not only by misdirecting PBP4, but also by destabilising PBP2a (Sewell and Brown, 2014).

(C) Other factors.

The cell wall is an important barrier that protects bacteria, such as *S. aureus*, from the external environment. Factors involved in cell wall synthesis and maintenance are also important for the resistance of *S. aureus* to antimicrobial agents (Scheffers and Pinho, 2005). Therefore, the cell wall could be a target of antibacterial sensitizers.

In *S. aureus*, lipoteichoic acid (LTA) is essential for growth and cell division (Gründling and Schneewind, 2007). Depletion of the synthase gene *ltaS* of LTA strongly resensitises MRSA to β-lactams *in vitro* (Therien et al., 2012). In addition, cell wall two-component signal transduction systems (TCSs) sense cell surface damage and trigger protective stress responses (Jordan et al., 2008). The sentinel *S. aureus* cell wall TCS is encoded by *vraSR* (Kuroda et al., 2003). Deletion of *vraSR* restored the efficacy of oxacillin in animal infection models against the community-acquired MRSA strain USA300 (Jo et al., 2011). FtsZ, a tubulin-like protein that forms the Z ring involved in cell division at the very beginning of cell wall synthesis, has also attracted much attention (Ferrer-González et al., 2021). PBP2a requires a chaperone, the peptidyl-prolyl cis-trans isomerase PrsA, to fold correctly (Jousselin et al., 2015). In *S. aureus*, staphyloxanthin is an unphosphorylated membrane saccharolipid that lends bacterial colonies a golden yellow colour (Liu et al., 2008). Inhibition of staphyloxanthin synthesis resulted in reduced PBP2a oligomerisation in the membrane and rendered MRSA cells susceptible to β-lactam antibiotics in a mouse infection model (Hennessy et al., 2016). Therefore, they may also be targets of antibacterial sensitizers.

#### 3.1.3 Mutation-dependent modification of PBP proteins

The criterion for methicillin resistance in *S. aureus* is an oxacillin MIC >2 mg/L according to the European Committee on Antimicrobial Susceptibility Testing (EUCAST) and oxacillin MIC ≥4 mg/L according to the Clinical and Laboratory Standards Institute (CLSI). The cefoxitin resistance criterion was also used for *S. aureus* and is defined by MIC >4 mg/L according to EUCAST and MIC ≥8 mg/L according to CLSI. Some *S. aureus* strains without the *mecA* gene to produce PBP2a are resistant to β-lactams because of mutations in genes encoding PBP2 and PBP4, albeit very rarely (Boonsiri et al., 2020). These strains were described as modified penicillin-binding protein *S. aureus* (MODSA) or methicillin-resistant lacking *mec* (MRLM).

### 3.2 Resistance to glycopeptide antibiotics

To date, glycopeptide antibiotics are the most frequently used drugs for the treatment of MRSA infections. However, there have been reports of reduced susceptibility and resistance to vancomycin globally since 1990 (Unni et al., 2021). The main mechanism of vancomycin resistance in MRSA is the acquisition of the *vanA* gene from enterococci (Gardete and Tomasz, 2014) changing the antibiotic target.

### 3.3 Resistance to macrolides, aminoglycosides, and oxazolidinones

The most common mechanism of resistance to macrolides in MRSA is the modification of the target site. Modification is carried out by the enzymes adenylyl-N-methyl transferase Erm (erythromycin ribosome methylation) and demethylating adenine 2058, which leads to resistance to all macrolides (Miklasińska-Majdanik, 2021). In addition, the expression of efflux pumps belongs to the ATP-binding cassette (ABC) efflux family, and inactivation of enzymes such as macrolide phosphotransferases may also contribute towards resistance to macrolides (Schmitz et al., 2000; Malbruny et al., 2002).

The mechanisms of resistance to aminoglycosides in MRSA include (1) synthesis of transferases (acetyltransferases, phosphotransferases, and nucleotidyl transferases) that modify the aminoglycoside molecule (Chandrakanth et al., 2008); and (2) lack of enzymes responsible for active transport of aminoglycosides into the bacterial cell (Melter and Radojevič, 2010).

Linezolid, an oxazolidinone that is highly active against MRSA, was introduced to treat MRSA after vancomycin-resistant strains were discovered (Quiles-Melero et al., 2013). The main mechanism of resistance to linezolid is mutations in a series of genes, which modifies the target site preventing linezolid action (Mendes et al., 2008; Hill et al., 2010; Liu et al., 2021).

### 3.4 Resistance to daptomycin

Daptomycin, a cyclic lipopeptide antibiotic, acts on the cytoplasmic membrane of *S. aureus,* causing loss of potassium ions from the cytoplasm. Resistance to this antibiotic has been attributed to mutations in various genes (*dltABCD*, *mprF*, and *rpoB*) that cause changes in membrane fluidity, cell wall thickness, and membrane charge (Kaatz et al., 2006).

### 3.5 Resistance to other kind of antibiotics

Expressing efflux pumps is a common resistance mechanism in MRSA against antibiotics such as tigecycline, tetracyclines, and fluoroquinolones. For tigecycline, resistant phenotypes emerged in 2008, and are considered to be caused by mutations in the *mepR* and *mepA* genes resulting in the overexpression of efflux pumps (Peterson, 2008). For tetracyclines, there is tet(K), which derives its energy from the proton pump and is classified as major facilitator super family (MFS) (Guay et al., 1993). Finally, for fluoroquinolones, NorA, NorB, NorC, and SdrM, all classified as MFS, are the main reasons for resistance (Xu et al., 2011). Table 2 summarized antibiotic resistant mechanisms in MRSA.

**TABLE 2:** Summary of antibiotic resistant mechanisms in MRSA.

Antibiotic class	Resistant mechanism
Penicillins and 4 generation of Cephalosporins	Penicillinase, production of PBP2a
Carbapenems	Development of PBP2a
Tetracyclines	Ribosomal methylation of binding sites, efflux pumps
Tigecyclines	Efflux pumps
Macrolides and clindamycin	Ribosomal methylation of binding sites, efflux pumps
Fluoroquinolones	Mutations in topoisomerase IV and DNA gyrase, efflux pumps
Rifampicin	Mutations in RNA polymerase gene
TMP/SMX	Mutations in DHPS and DHFR
Aminoglycosides	Aminoglycoside degradation enzymes
Daptomycin	Electrostatic repulsion through increase to the cell-surface charge
Vancomycin	VRSA: altered structure of peptidoglycan precursors from D-Ala-D-Ala to D-Ala-D-Lac; VISA: increased production of peptidoglycan, thicker cell wall, decoy D-Ala-D-Ala dipeptides on cell surface
Linezolid	Mutations to the 23S rRNA, altering required modifications to the 23S rRNA, mutations to the 50S ribosomal L3 protein

Abbreviations: PBP, penicillin binding protein; TMP/SMX, trimethoprim-sulfamethoxazole; DHPS, dihydropteroate synthase; DHFR, dihydrofolate reductase.

## 4 Antibacterial sensitizers

Antibiotic sensitizers are also called “antibiotic adjuvants” or “antibiotic potentiators” (Wright, 2016). They have little or no antibiotic activity, but can enhance the antimicrobial action of a drug when combined with antibiotics. From the perspective of drug discovery, this strategy offers the advantage of overcoming antibiotic resistance.

### 4.1 Antibacterial sensitizers targeting beta-lactamases and other hydrolytic enzymes

To date, β-lactamase inhibitors have been successfully used in clinical trials, including clavulanic acid, sulbactam, tazobactam, avibactam, and vaborbactam (Yahav et al., 2020). The most commonly reported enzyme inhibitors in plants are β-lactamase inhibitors.

For example, epigallocatechin gallate (EGCG), the major catechin present in *Camellia sinensis* (L.) Kuntze tea leaves, has been reported to potentiate the activity of β-lactams against MRSA (Abreu et al., 2012). A possible mechanism is the inhibition of β-lactamases in *S. aureus* (Zhao et al., 2002) and interference with the integrity of the cell wall through direct binding to PG (Zhao et al., 2001). Baicalein, the active constituent of *Scutellaria baicalensis*, combined with penicillin, showed potent synergistic activity against penicillinase-producing MRSA *in vitro* (Qian et al., 2015). Alkaloid compounds from *Cienfuegosia digitata* Cav. Combined with β-lactams showed strong activity against MRSA, probably through the inhibition of β-lactamase (Konaté et al., 2012). Tellimagrandin I, isolated from rose red petals, showed a marked reduction in β-lactams against MRSA. The same effect was observed for rugosin B, which is also a constituent of rose red petals (Shiota et al., 2000). A possible mechanism is the suppression of β-lactamase activity to some extent (Shiota et al., 2004). A combination of ampicillin with 5-O-methylglovanon from *Glycosmis* showed activity comparable to that of clavulanic acid in combination with ampicillin (Zhou et al., 2011). The mechanism of 5-O-methylglovanon involves the inhibition of β-lactamases (Obiang-Obounou and Jang, 2011). SB-202742 isolated from *Spondias mombin* showed strong inhibitory activity against β-lactamase and can also sensitise MRSA to β-lactams (Coates et al., 1994). Activity against *K. pneumoniae* ATCC 700603 was also observed for 2-methoxy chrysophanol from *Clutia myricoides*in combination with third-generation cephalosporin antibiotics through the inhibition of extended-spectrum β-lactamases (ESBL), with marked enlargement of inhibition zones (>5 mm) (Elfaky et al., 2020).

Besides β-lactamases, there are other hydrolytic enzymes in MRSA that hydrolyse other types of antibiotics. Aranorosin isolated from *Gymnascella aurantiaca* inhibited the growth of a MRSA strain in the presence of arbekacin and was confirmed to act by inhibiting a bifunctional aminoglycoside-modifying enzyme (Labby and Garneau-Tsodikova, 2013). Inhibitors of chloramphenicol acetyltransferase have not been reported in the past 20 years Table 3 presents an overview of antibacterial sensitizers that target β-lactamases and other hydrolytic enzymes.

**TABLE 3:** The compounds from natural products that affect beta-lactamases and other hydrolytic enzymes.

Name	Source	Mechanisms	Ref
EGCG	Tea *Camellia sinensis (*L.*) Kuntze* leaves	Inhibition of beta-lactamases	Zhao et al. (2002)
Baicalein	*Scutellaria baicalensis*	Inhibition of penicillinase	Qian et al. (2015)
Alkaloid compounds	*Cienfuegosia digitata* Cav	Inhibition of beta-lactamases	Konaté et al. (2012)
Tellimagrandin I and rugosin B	rose red petals	Inhibition of beta-lactamases	Shiota et al. (2000)
			Shiota et al. (2004)
5-O-Methylglovanon	Glycosmis plant	Inhibition of beta-lactamases	Obiang-Obounou and Jang (2011)
SB-202742	*Spondias mombin*	Inhibition of beta-lactamases	Coates et al. (1994)
2-methoxy chrysophanol	*Clutia myricoides*	inhibiting Extended-Spectrum beta-lactamases (ESBL)	Elfaky et al. (2020)
Aranorosin	*Gymnascella aurantiaca*	inhibiting the bifunctional aminoglycoside-modifying enzyme	Labby and Garneau-Tsodikova (2013)

### 4.2 Antibacterial sensitizers targeting PBP2a and its Co-Factors

#### 4.2.1 Antibacterial sensitizers targeting PBP2a

MRSA contains an SCCmec box encoding PBP2a by horizontal transfer which has a low affinity to most beta-lactam antibiotics (Peacock and Paterson, 2015). Acquiring the PBP2a protein is the most important mechanism underlying MRSA resistance to β-lactams. Numerous studies have indicated that small molecules from herbal medicines combined with β-lactams have a synergistic effect, and their target is PBP2a.

For example, the synergistic effect of oxacillin and morin, a natural pentahydroxy flavonol, against MRSA occurs by reducing the production of PBP2a, encoded by the *mecA* gene (Mun et al., 2015). The ursolic/oleanolic acids extracted from the leaves of Shea butter trees exhibited synergistic effects in combination with ampicillin/oxacillin against MRSA. The mechanism of reversion of the MRSA phenotype involves the ability to separate PBP2a from the cleavage site of the spacer, which interferes with the synthesis of PGs (Catteau et al., 2017). Another report showed that ampicillin/sulbactam combined with EGCG from tea catechins reversed ampicillin/sulbactam resistance against MRSA in a dose-dependent manner through the direct binding of PGs (Hu et al., 2001). This is consistent with a previous report that oxacillin and EGCG elicit synergistic effects against MRSA by reducing the production of PBP2a or its gene expression (Shiota et al., 1999). The synergistic antimicrobial effect of oxacillin and corilagin/tellimagrandin I demonstrated that corilagin and tellimagrandin I play an important role in inhibiting the activity of PBP2a rather than its production of PBP2a (Shiota et al., 2004). Baicalein, a flavone isolated from this herb, showed asynergistic effect in combination with benzylpenicillin against MRSA in a dose-dependent manner. The mechanism of action of baicalein is related to the inhibition of PBP2a or damage to PG (Liu et al., 2000). 2,3,3-Trimethyl-octane and benzoic acid from the methanolic bark extract of *Toxicodendron vernicifluum* have been reported to metabolise novel bacterial topoisomerase inhibitors (NBTI) and PBP2a, resulting in excellent antibacterial activity (Saravanakumar et al., 2019). Table 4 presents an overview of antibacterial sensitizers that inhibit PBP2a. (Table 4).

**TABLE 4:** The compounds from natural products that inhibit PBP2a.

Name	Source	Mechanisms	Ref
Morin	Flavonol	Reducing the production of PBP2a	Mun et al. (2015)
Ursolic/Oleanolic acids	leaves of shea butter trees	Separating PBP2a from the cleavage site of the spacer	Catteau et al. (2017)
EGCG	Tea *Camellia sinensis (*L.*) Kuntze* leaves	Binding of peptidoglycans, reducing the production of PBP2a or its gene expression	Hu et al., 2001, Shiota et al., 1999
Corilagin/Tellimagrandin I		Inhibiting the activity of PBP2a	Shiota et al. (2004)
Baicalein		Inhibition of PBP2a or damage of peptidoglycans	Liu et al. (2000)
2,3,3-trimethyl-Octane and benzoic	*Toxicodendron vernicifluum*	Metabolizing PBP2a	Saravanakumar et al. (2019)

#### 4.2.2 Antibacterial sensitizers targeting factors involved in *S. aureus* cell wall biogenesis

Several small-molecule inhibitors have been discovered in the field of Mur enzymes in the last decade. However, these inhibitors are mostly derived from chemical synthesis and there are very few small molecules that are derived from natural products (Hrast et al., 2014).

#### 4.2.3 Antibacterial sensitizers targeting factors involved in *S. aureus* wall teichoic acid synthesis

Numerous studies have reported various molecules targeting Tar enzymes, such as tunicamycin and ticlopidine as tarO inhibitors (Campbell et al., 2011; Farha et al., 2013) as well as targosey and clomiphene as tarG inhibitors (Swoboda et al., 2009; Farha et al., 2015). However, Tar enzyme inhibitors from natural plants have not been reported in the last 20 years.

#### 4.2.4 Antibacterial sensitizers targeting other factors

Compound 1771 and Congo red are the only two inhibitors of *ltaS* (Richter et al., 2013; Vickery et al., 2018). Novel FtsZ inhibitors, 1-methylquinolinium iodide derivative and quinuclidine 1, have been reported to possess strong and synergistic antibacterial activity against MRSA when combined with β-lactams (Chan et al., 2015; Fang et al., 2018).

### 4.3 Antibacterial sensitizers targeting efflux pumps

Baicalein has previously been shown to significantly reverse ciprofloxacin resistance in MRSA, possibly by inhibiting the NorA efflux pump *in vitro* (Chan et al., 2011). 5'-Methoxyhydnocarpin (5'-MHC) was previously reported to have no antimicrobial activity but strongly potentiated the action of berberine and other NorA substrates, such as ethidium bromide, against *S. aureus* (Stermitz et al., 2000a). Piperine, in combination with ciprofloxacin, markedly reduced the MICs of ciprofloxacin against MRSA. The enhanced accumulation and decreased efflux of ethidium bromide in the wild type and mutant (CIPr-1) strains in the presence of piperine suggests its involvement in the inhibition of NorA pumps (Khan et al., 2006). The ethanolic extract of *Persea lingue*, kaempferol-3-O-alpha-L-(2, 4-bis-E-p-coumaroyl) rhamnoside, when combined with ciprofloxacin, resulted in a synergistic effect with antimicrobial activity against a NorA overexpressor increasing to 8-fold higher than the activity of ciprofloxacin monotherapy. However, a similar trend was not observed against a *norA* deletion mutant (Holler et al., 2012). Indirubin was isolated from a chloroform extract of *W. tinctoria R. Br*. leaves, capsaicin from chilli peppers, and carnosol/carnosic acid from *Rosmarinus officinalis* synergistically potentiated the activity of ciprofloxacin and erythromycin, probably by inhibiting the NorA efflux pump (Oluwatuyi et al., 2004; Ponnusamy et al., 2010; Kalia et al., 2012). Silybin, a flavonolignan component of milk thistle seed extract, has been shown to restore the sensitivity of MRSA 41577 to antibiotics. The possible mechanism was related to the reduction in the expression of the quinolone resistance protein NorA (*norA*) and quaternary ammonium resistance protein A/B (*qacA/B*) efflux genes in MRSA through reverse transcription-quantitative polymerase chain reaction analysis (Wang et al., 2018). Many herbal compounds can inhibit the NorA pump in *S. aureus* to restore bacterial resistance to antibiotics. Table 5 summarises the compounds from plants that inhibit the NorA pump.

**TABLE 5:** The compounds from natural products that inhibit NorA pump.

Name	Source	Mechanism	Ref
5-methoxyhydnocarpin (5-MHC)	*Hydnocarpus wightiana*	Inhibition of NorA pump	Stermitz et al. (2000a)
Piperine	*Piper nigrum*		Khan et al. (2006)
Kaempferol-3-O-α-L-(2,4-bis-E-p-coumaroyl) rhamnoside	*Persea lingue*		Holler et al. (2012)
Capsaicin	*chili peppers*		Kalia et al. (2012)
Carnosol and carnosic acid	*Rosmarinus officinalis*		Oluwatuyi et al. (2004)
Indirubin	*Wrightia tinctoria*		Ponnusamy et al. (2010)
Porphyrin pheophorbide A	*Berberis species*		Stermitz et al. (2000b)
Sarothrin	*Alkanna Orientalis*		Bame et al. (2013)
Orizabin XIX	*Mexican morning glory species*		Pereda-Miranda et al. (2006)
Totarol	*Chamaecyparis nootkatensis*		Smith et al. (2007)
Terpenoid	*Euphorbia hirta*		Perumal and Mahmud (2013)
Arylbenzofuran aldehyde (Spinosan A)	*Dalea spinose*		Belofsky et al. (2006)
Flavanoid/phenolic compounds	*Dalea versicolor*		Belofsky et al. (2004)

Except for NorA, the proton-driven multidrug efflux pump LmrS actively exports structurally distinct antimicrobials. Cumin inhibited the growth of *S. aureus* and LmrS ethidium transport in a dose-dependent manner (Kakarla et al., 2017). In a previous study, EGCG also enhanced oxytetracycline activity against MRSA, and the MIC of oxytetracycline was reduced 4–12-fold when it was combined with EGCG and the possible mechanism is inhibition of the efflux pump tet(K) (Novy et al., 2013). Baicalein also inhibits tet(K) pumps to potentiate tetracycline antibiotics (Fujita et al., 2005). Diosmetin showed a strong synergistic effect against MRSA strains when combined with antibiotics. The probable mechanism may be the inhibition of efflux pumps (Wang et al., 2014).

### 4.4 Antibacterial sensitizers targeting the cell membrane and other targets

Gallic acid-grafted chitosans (I), which have the highest gallic acid content, exhibited synergistic effects when combined with β-lactams by damaging the cell membrane (Lee et al., 2014). The natural polyphenolic flavonoid apigenin has been found to enhance the activity of ampicillin and ceftriaxone against MRSA. Apigenin may damage the MRSA cytoplasmic membrane and cause subsequent leakage of intracellular constituents (Akilandeswari and Ruckmani, 2016). In addition, many compounds have synergistic effects when combined with antibiotics and other mechanisms. It has been found that lipoic acid derivatives exhibit excellent activity against multidrug-resistant *S. aureus* with no cytotoxicity and sensitise fluoroquinolones towards fluoroquinolone-resistant methicillin-resistant *S. aureus* strains, with DNA gyrase B as the likely molecular target, as determined by molecular dynamics (MD) simulations (Panjla et al., 2019). C-geranylated flavonoids from *Paulownia tomentosa* fruits have been reported to reverse the antibiotic effects of oxacillin (Navrátilová et al., 2016). It is mechanism was not determined.

## 5 Conclusion and perspectives

**FIGURE 2:**
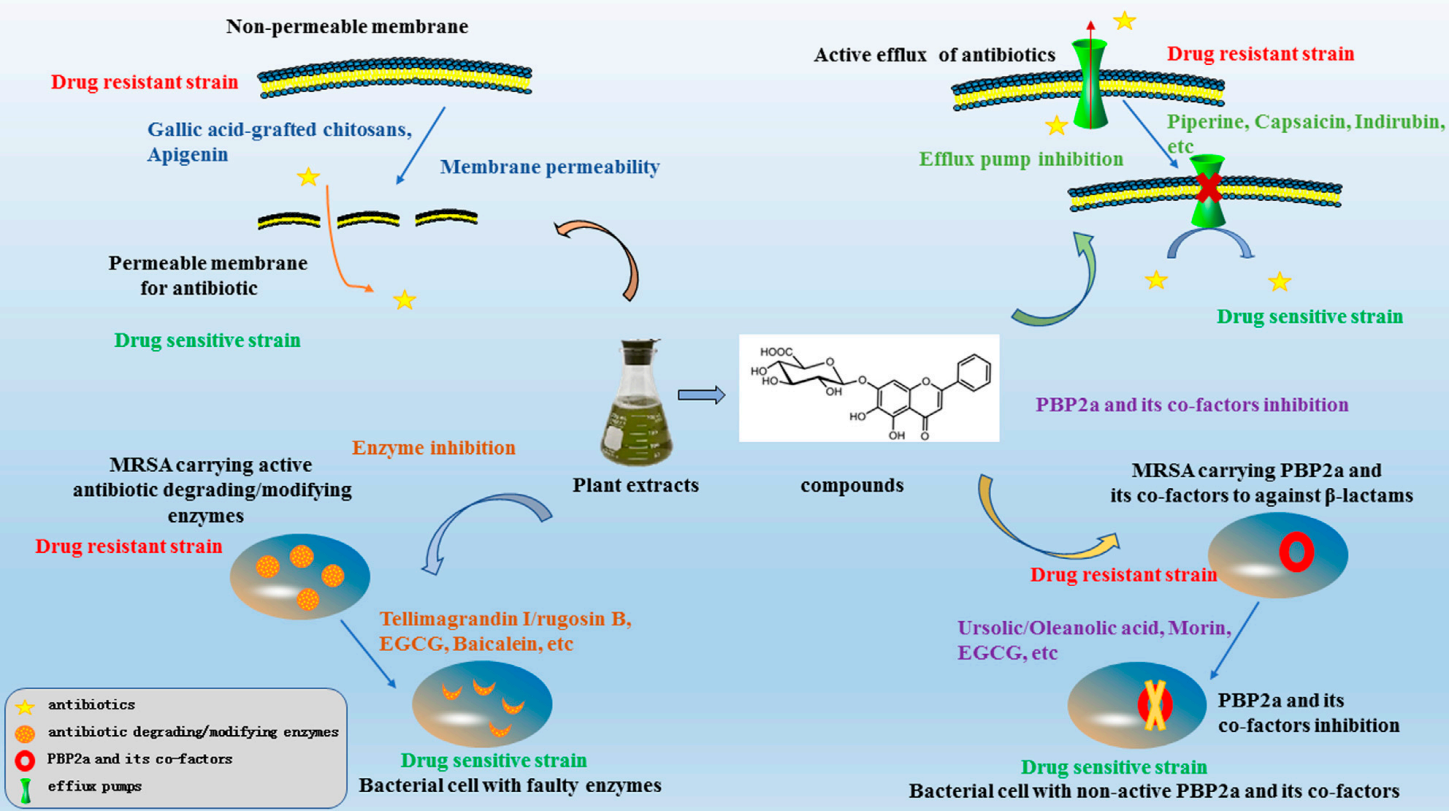
The mechanisms of antibacterial sensitizers against MRSA.

In this review, we have summarised the mechanisms of resistance in MRSA and investigations regarding antibacterial sensitizers from natural products and their possible mechanisms over the last two decades. The main mechanisms of antibacterial sensitizers were showed in Figure 2. There still were a lot of reports showed that not only isolated pure natural products but also extracts from plants exhibited strong synergy effects with antibiotics. So antibacterial sensitizers and antimicrobial drugs combinations have revealed promising results in the fight against multidrug-resistant bacteria.

The rich chemical diversity of plants makes them a renewable and attractive potential source of antimicrobial sensitizers. Numerous studies have demonstrated the therapeutic potential of phytochemical products as antibiotics. However, there are no reports on the clinical applications of natural-product-derived antibacterial sensitizers. The reasons may be as follows: (1) the chemical complexity of plant extracts, often undocumented toxicity, poor water solubility, and lack of standardisation (Cos et al., 2006; Spížek et al., 2010), (2) the uncertainty in the chemical composition of natural plants, (3) the inherent time consumption of working with natural products, and (4) additional limitations such as the costs of collection, extraction, and isolation (Harvey, 2008). Therefore, there is still a long way to go before antimicrobial sensitizers from natural plants can be used in clinical settings.

Because plant-derived antibacterial sensitizers faced so many problems, there are several methods that may be used to solve them. On one hand, we can use some new techniques to discover new antibacterial sensitizers and their mechanisms of action. (1) With the development of genomics, proteomics, and metabolomics, they create a platform for the screening of antibacterial sensitizers and the study of their pharmacological effect and mechanism. (2) Molecular docking and simulation studies can also help researchers to quickly screen the potential candidate drugs based on the resistant targets of MRSA, and understand the possible relationship of antibacterial sensitizers and targets. (3) Bioassay-guiding isolation of active products is crucial to study the synergistic mechanism in-depth. On the other hand, we can use some approaches to improve the sensitizing effect of existing antibacterial sensitizers. (1) Improve the quality standard of active compounds when they were extracted and separated. For example, the picking season and origin of the herb, the process of extraction and the using of solvent, *etc.*, needed to be strictly controlled. (2) Optimizing the combination ratio and proper dosage regimen of the combinations are still important. (3) Furthermore, comparable pharmacokinetic profiles of both drugs can bring more effective synergistic combinations, so future studies could focus on pharmacokinetic behaviours of antibacterial sensitizers and antibiotics.

## References

[B1] Abreu A. C. McBain A. J. Simões M. (2012). Plants as sources of new antimicrobials and resistance-modifying agents. Nat. Prod. Rep. 29, 1007–1021. 10.1039/c2np20035j 22786554

[B2] Akilandeswari K. Ruckmani K. (2016). Synergistic antibacterial effect of apigenin with β-lactam antibiotics and modulation of bacterial resistance by a possible membrane effect against methicillin resistant *Staphylococcus aureus* . Cell Mol. Biol. (Noisy-le-grand) 62, 74–82. 10.14715/cmb/2016.62.14.13 28145860

[B3] Bame J. R. Graf T. N. Junio H. A. Bussey R. O. Jarmusch S. A. El-Elimat T. (2013). Sarothrin from Alkanna orientalis is an antimicrobial agent and efflux pump inhibitor. Planta Med. 79, 327–329. 10.1055/s-0032-1328259 23468310 PMC4527991

[B4] Belofsky G. Carreno R. Lewis K. Ball A. Casadei G. Tegos G. P. (2006). Metabolites of the "smoke tree", Dalea spinosa, potentiate antibiotic activity against multidrug-resistant *Staphylococcus aureus* . Dalea spinosa, potentiate Antibiot. Act. against multidrug-resistant Staphylococcus Aureus. *J. Nat. Prod* . 69, 261–264. 10.1021/np058057s 16499327

[B5] Belofsky G. Percivill D. Lewis K. Tegos G. P. Ekart J. (2004). Phenolic metabolites of Dalea versicolor that enhance antibiotic activity against model pathogenic bacteria. J. Nat. Prod. 67, 481–484. 10.1021/np030409c 15043439

[B6] Boonsiri T. Watanabe S. Tan X. E. Thitiananpakorn K. Narimatsu R. Sasaki K. (2020). Identification and characterization of mutations responsible for the β-lactam resistance in oxacillin-susceptible mecA-positive *Staphylococcus aureus* . Sci. Rep. 10, 16907. 10.1038/s41598-020-73796-5 33037239 PMC7547103

[B7] Brown S. Xia G. Luhachack L. G. Campbell J. Meredith T. C. Chen C. (2012). Methicillin resistance in *Staphylococcus aureus* requires glycosylated wall teichoic acids. Proc. Natl. Acad. Sci. U. S. A. 109, 18909–18914. 10.1073/pnas.1209126109 23027967 PMC3503181

[B8] Bush K. Bradford P. A. (2020). Epidemiology of β-lactamase-producing pathogens. Clin. Microbiol. Rev. 33, 000477–e119. 10.1128/CMR.00047-19 PMC7048014 32102899

[B9] Bush K. Jacoby G. A. (2010). Updated functional classification of β-Lactamases. Antimicrob. Agents Chemother. 54, 969–976. 10.1128/AAC.01009-09 19995920 PMC2825993

[B10] Campbell J. Singh A. K. Santa Maria J. P. Kim Y. Brown S. Swoboda J. G. (2011). Synthetic lethal compound combinations reveal a fundamental connection between wall teichoic acid and peptidoglycan biosyntheses in *Staphylococcus aureus* . ACS Chem. Biol. 6, 106–116. 10.1021/cb100269f 20961110 PMC3025082

[B11] Catteau L. Reichmann N. T. Olson J. Pinho M. G. Nizet V. Van Bambeke F. (2017). Synergy between ursolic and oleanolic acids from vitellaria paradoxa leaf extract and β-lactams against methicillin-resistant *Staphylococcus aureus*: *In vitro* and *in vivo* activity and underlying mechanisms. Molecules 22, E2245. 10.3390/molecules22122245 PMC6149719 29258194

[B12] Chan B. C. L. Ip M. Lau C. B. S. Lui S. L. Jolivalt C. Ganem-Elbaz C. (2011). Synergistic effects of baicalein with ciprofloxacin against NorA over-expressed methicillin-resistant *Staphylococcus aureus* (MRSA) and inhibition of MRSA pyruvate kinase. *J. Ethnopharmacol*137 137, 767–773. 10.1016/j.jep.2011.06.039 21782012

[B13] Chan F. Y. Sun N. Leung Y. C. Wong K. Y. (2015). Antimicrobial activity of a quinuclidine-based FtsZ inhibitor and its synergistic potential with β-lactam antibiotics. J. Antibiot. (Tokyo) 68, 253–258. 10.1038/ja.2014.140 25293977

[B14] Chandrakanth R. K. Raju S. Patil S. A. (2008). Aminoglycoside-resistance mechanisms in multidrug-resistant *Staphylococcus aureus* clinical isolates. Curr. Microbiol. 56, 558–562. 10.1007/s00284-008-9123-y 18320273

[B15] Coates N. J. Gilpin M. L. Gwynn M. N. Lewis D. E. Milner P. H. Spear S. R. (1994). SB-202742, a novel beta-lactamase inhibitor isolated from Spondias mombin. J. Nat. Prod. 57, 654–657. 10.1021/np50107a016 8064298

[B16] Cos P. Vlietinck A. J. Berghe D. V. Maes L. (2006). Anti-infective potential of natural products: How to develop a stronger *in vitro* “proof-of-concept. J. Ethnopharmacol. 106, 290–302. 10.1016/j.jep.2006.04.003 16698208

[B17] de Oliveira Santos J. V. da Costa Júnior S. D. de Fátima Ramos dos Santos Medeiros S. M. Cavalcanti I. D. L. de Souza J. B. Coriolano D. L. (2022). Panorama of bacterial infections caused by epidemic resistant strains. Curr. Microbiol. 79, 175. 10.1007/s00284-022-02875-9 35488983 PMC9055366

[B18] Elfaky M. A. El-Halawany A. M. Koshak A. E. Alshali K. Z. El-Araby M. E. Khayat M. T. (2020). Bioassay guided isolation and docking studies of a potential β-Lactamase inhibitor from Clutia myricoides. Molecules 25, E2566. 10.3390/molecules25112566 PMC7321312 32486455

[B19] Fang Z. Ban L. Li Y. Yuan W. Liu Z. Liu T. (2018). A quinoline-based FtsZ inhibitor for the study of antimicrobial activity and synergistic effects with β-lactam antibiotics. J. Pharmacol. Sci. 137, 283–289. 10.1016/j.jphs.2018.07.005 30057277

[B20] Farha M. A. Czarny T. L. Myers C. L. Worrall L. J. French S. Conrady D. G. (2015). Antagonism screen for inhibitors of bacterial cell wall biogenesis uncovers an inhibitor of undecaprenyl diphosphate synthase. Proc. Natl. Acad. Sci. U. S. A. 112, 11048–11053. 10.1073/pnas.1511751112 26283394 PMC4568241

[B21] Farha M. A. Leung A. Sewell E. W. D’Elia M. A. Allison S. E. Ejim L. (2013). Inhibition of WTA synthesis blocks the cooperative action of PBPs and sensitizes MRSA to β-lactams. ACS Chem. Biol. 8, 226–233. 10.1021/cb300413m 23062620 PMC3552485

[B22] Ferrer-González E. Huh H. Al-Tameemi H. M. Boyd J. M. Lee S.-H. Pilch D. S. (2021). Impact of FtsZ inhibition on the localization of the Penicillin binding proteins in Methicillin-resistant *Staphylococcus aureus* . J. Bacteriol. 203, e0020421. 10.1128/JB.00204-21 34031040 PMC8297533

[B23] Fujita M. Shiota S. Kuroda T. Hatano T. Yoshida T. Mizushima T. (2005). Remarkable synergies between baicalein and tetracycline, and baicalein and beta-lactams against methicillin-resistant *Staphylococcus aureus* . Microbiol. Immunol. 49, 391–396. 10.1111/j.1348-0421.2005.tb03732.x 15840965

[B24] Gardete S. Tomasz A. (2014). Mechanisms of vancomycin resistance in *Staphylococcus aureus* . J. Clin. Invest. 124, 2836–2840. 10.1172/JCI68834 24983424 PMC4071404

[B25] Gründling A. Schneewind O. (2007). Genes required for glycolipid synthesis and lipoteichoic acid anchoring in *Staphylococcus aureus* . J. Bacteriol. 189, 2521–2530. 10.1128/JB.01683-06 17209021 PMC1899383

[B26] Guay G. G. Khan S. A. Rothstein D. M. (1993). The tet(K) gene of plasmid pT181 of *Staphylococcus aureus* encodes an efflux protein that contains 14 transmembrane helices. Plasmid 30, 163–166. 10.1006/plas.1993.1045 8234490

[B27] Harvey A. L. (2008). Natural products in drug discovery. Drug Discov. Today 13, 894–901. 10.1016/j.drudis.2008.07.004 18691670

[B28] Hennessy E. Adams C. Reen F. J. O’Gara F. (2016). Is there potential for repurposing statins as novel antimicrobials? Antimicrob. Agents Chemother. 60, 5111–5121. 10.1128/AAC.00192-16 27324773 PMC4997871

[B29] Hill R. L. R. Kearns A. M. Nash J. North S. E. Pike R. Newson T. (2010). Linezolid-resistant ST36 methicillin-resistant *Staphylococcus aureus* associated with prolonged linezolid treatment in two paediatric cystic fibrosis patients. J. Antimicrob. Chemother. 65, 442–445. 10.1093/jac/dkp494 20089543

[B30] Holler J. G. Christensen S. B. Slotved H. C. Rasmussen H. B. Gúzman A. Olsen C. E. (2012). Novel inhibitory activity of the *Staphylococcus aureus* NorA efflux pump by a kaempferol rhamnoside isolated from Persea lingue Nees. J. Antimicrob. Chemother. 67, 1138–1144. 10.1093/jac/dks005 22311936

[B31] Hrast M. Sosič I. Sink R. Gobec S. (2014). Inhibitors of the peptidoglycan biosynthesis enzymes MurA-F. Bioorg Chem. 55, 2–15. 10.1016/j.bioorg.2014.03.008 24755374

[B32] Hu Z. Q. Zhao W. H. Hara Y. Shimamura T. (2001). Epigallocatechin gallate synergy with ampicillin/sulbactam against 28 clinical isolates of methicillin-resistant *Staphylococcus aureus* . J. Antimicrob. Chemother. 48, 361–364. 10.1093/jac/48.3.361 11533000

[B33] Jo D. S. Montgomery C. P. Yin S. Boyle-Vavra S. Daum R. S. (2011). Improved oxacillin treatment outcomes in experimental skin and lung infection by a methicillin-resistant *Staphylococcus aureus* isolate with a vraSR operon deletion. Antimicrob. Agents Chemother. 55, 2818–2823. 10.1128/AAC.01704-10 21383093 PMC3101413

[B34] Jordan S. Hutchings M. I. Mascher T. (2008). Cell envelope stress response in Gram-positive bacteria. FEMS Microbiol. Rev. 32, 107–146. 10.1111/j.1574-6976.2007.00091.x 18173394

[B35] Jousselin A. Manzano C. Biette A. Reed P. Pinho M. G. Rosato A. E. (2015). The *Staphylococcus aureus* Chaperone PrsA is a new auxiliary factor of Oxacillin resistance affecting Penicillin-binding protein 2A. Antimicrob. Agents Chemother. 60, 1656–1666. 10.1128/AAC.02333-15 26711778 PMC4775990

[B36] Kaatz G. W. Lundstrom T. S. Seo S. M. (2006). Mechanisms of daptomycin resistance in *Staphylococcus aureus* . Int. J. Antimicrob. Agents 28, 280–287. 10.1016/j.ijantimicag.2006.05.030 16963232

[B37] Kakarla P. Floyd J. Mukherjee M. Devireddy A. R. Inupakutika M. A. Ranweera I. (2017). Inhibition of the multidrug efflux pump LmrS from *Staphylococcus aureus* by cumin spice Cuminum cyminum. Arch. Microbiol. 199, 465–474. 10.1007/s00203-016-1314-5 27830269

[B38] Kalia N. P. Mahajan P. Mehra R. Nargotra A. Sharma J. P. Koul S. (2012). Capsaicin, a novel inhibitor of the NorA efflux pump, reduces the intracellular invasion of *Staphylococcus aureus* . J. Antimicrob. Chemother. 67, 2401–2408. 10.1093/jac/dks232 22807321

[B39] Khan I. A. Mirza Z. M. Kumar A. Verma V. Qazi G. N. (2006). Piperine, a phytochemical potentiator of ciprofloxacin against *Staphylococcus aureus* . Antimicrob. Agents Chemother. 50, 810–812. 10.1128/AAC.50.2.810-812.2006 16436753 PMC1366922

[B40] Konaté K. Mavoungou J. F. Lepengué A. N. Aworet-Samseny R. R. Hilou A. Souza A. (2012). Antibacterial activity against β-lactamase producing methicillin and ampicillin-resistants *Staphylococcus aureus*: Fractional inhibitory concentration index (FICI) determination. Ann. Clin. Microbiol. Antimicrob. 11, 18. 10.1186/1476-0711-11-18 22716026 PMC3464800

[B42] Kuroda M. Kuroda H. Oshima T. Takeuchi F. Mori H. Hiramatsu K. (2003). Two-component system VraSR positively modulates the regulation of cell-wall biosynthesis pathway in *Staphylococcus aureus* . Mol. Microbiol. 49, 807–821. 10.1046/j.1365-2958.2003.03599.x 12864861

[B43] Labby K. J. Garneau-Tsodikova S. (2013). Strategies to overcome the action of aminoglycoside-modifying enzymes for treating resistant bacterial infections. Future Med. Chem. 5, 1285–1309. 10.4155/fmc.13.80 23859208 PMC3819198

[B44] Lee D. S. Eom S. H. Kim Y. M. Kim H. S. Yim M. J. Lee S. H. (2014). Antibacterial and synergic effects of gallic acid-grafted-chitosan with β-lactams against methicillin-resistant *Staphylococcus aureus* (MRSA). Can. J. Microbiol. 60, 629–638. 10.1139/cjm-2014-0286 25216286

[B45] Liang M. Ge X. Xua H. Ma K. Zhang W. Zan Y. (2022). Phytochemicals with activity against methicillin-resistant *Staphylococcus aureus* . Phytomedicine 100, 154073. 10.1016/j.phymed.2022.154073 35397285

[B46] Liu C. I. Liu G. Y. Song Y. Yin F. Hensler M. E. Jeng W. Y. (2008). A cholesterol biosynthesis inhibitor blocks *Staphylococcus aureus* virulence. Science 319, 1391–1394. 10.1126/science.1153018 18276850 PMC2747771

[B47] Liu I. X. Durham D. G. Richards R. M. (2000). Baicalin synergy with beta-lactam antibiotics against methicillin-resistant *Staphylococcus aureus* and other beta-lactam-resistant strains of *S. aureus* . J. Pharm. Pharmacol. 52, 361–366. 10.1211/0022357001773922 10757427

[B48] Liu W. T. Chen E. Z. Yang L. Peng C. Wang Q. Xu Z. (2021). Emerging resistance mechanisms for 4 types of common anti-MRSA antibiotics in *Staphylococcus aureus*: A comprehensive review. Microb. Pathog. 156, 104915. 10.1016/j.micpath.2021.104915 33930416

[B49] Luo R. Zhao L. Du P. Luo H. Ren X. Lu P. (2020). Characterization of an Oxacillin-Susceptible mecA-positive *Staphylococcus aureus* isolate from an imported meat product. Microb. Drug Resist 26, 89–93. 10.1089/mdr.2018.0211 31424352

[B50] Malbruny B. Canu A. Bozdogan B. Fantin B. Zarrouk V. Dutka-Malen S. (2002). Resistance to quinupristin-dalfopristin due to mutation of L22 ribosomal protein in *Staphylococcus aureus* . Antimicrob. Agents Chemother. 46, 2200–2207. 10.1128/AAC.46.7.2200-2207.2002 12069975 PMC127308

[B51] Melter O. Radojevič B. (2010). Small colony variants of Staphylococcus aureus--review. Folia Microbiol. 6, 548–558. 10.1007/s12223-010-0089-3 21253898

[B52] Mendes R. E. Deshpande L. M. Castanheira M. DiPersio J. Saubolle M. A. Jones R. N. (2008). First report of cfr-mediated resistance to linezolid in human staphylococcal clinical isolates recovered in the United States. Antimicrob. Agents Chemother. 52, 2244–2246. 10.1128/AAC.00231-08 18391032 PMC2415768

[B53] Miklasińska-Majdanik M. (2021). Mechanisms of resistance to macrolide antibiotics among *Staphylococcus aureus* . Antibiot. (Basel) 10, 1406. 10.3390/antibiotics10111406 PMC8615237 34827344

[B54] Mlynarczyk-Bonikowska B. Kowalewski C. Krolak-Ulinska A. Marusza W. (2022). Molecular mechanisms of drug resistance in *Staphylococcus aureus* . Int. J. Mol. Sci. 23, 8088. 10.3390/ijms23158088 35897667 PMC9332259

[B55] Mun S. H. Lee Y. S. Han S. H. Lee S. W. Cha S. W. Kim S. (2015). *In vitro* potential effect of Morin in the combination with β-Lactam antibiotics against Methicillin-resistant *Staphylococcus aureus* . Foodborne Pathog. Dis. 12, 545–550. 10.1089/fpd.2014.1923 26067230

[B56] Navrátilová A. Nešuta O. Vančatová I. Čížek A. Varela-M, R. E. López-Abán J. (2016). C-Geranylated flavonoids from Paulownia tomentosa fruits with antimicrobial potential and synergistic activity with antibiotics. Pharm. Biol. 54, 1398–1407. 10.3109/13880209.2015.1103755 26789098

[B57] Novy P. Rondevaldova J. Kourimska L. Kokoska L. (2013). Synergistic interactions of epigallocatechin gallate and oxytetracycline against various drug resistant *Staphylococcus aureus* strains *in vitro* . Phytomedicine 20, 432–435. 10.1016/j.phymed.2012.12.010 23485046

[B58] Obiang-Obounou B. W. Jang Y. P. (2011). Enriching modern pharmacotherapy through synergy assessment for the combination of natural products and synthetic drugs. Arch. Pharm. Res. 34, 1579–1581. 10.1007/s12272-011-1000-8 22076755

[B59] Odds F. C. (2003). Synergy, antagonism, and what the chequerboard puts between them. J. Antimicrob. Chemother. 52, 1. 10.1093/jac/dkg301 12805255

[B60] Oluwatuyi M. Kaatz G. W. Gibbons S. (2004). Antibacterial and resistance modifying activity of Rosmarinus officinalis. Phytochemistry 65, 3249–3254. 10.1016/j.phytochem.2004.10.009 15561190

[B61] Panjla A. Kaul G. Shukla M. Tripathi S. Nair N. N. Chopra S. (2019). A novel molecular scaffold resensitizes multidrug-resistant *S. aureus* to fluoroquinolones. Chem. Commun. (Camb) 55, 8599–8602. 10.1039/c9cc03001h 31276129

[B62] Pasquina L. W. Santa Maria J. P. Walker S. (2013). Teichoic acid biosynthesis as an antibiotic target. Curr. Opin. Microbiol. 16, 531–537. 10.1016/j.mib.2013.06.014 23916223 PMC3834221

[B63] Peacock S. J. Paterson G. K. (2015). Mechanisms of methicillin resistance in *Staphylococcus aureus* . Annu. Rev. Biochem. 84, 577–601. 10.1146/annurev-biochem-060614-034516 26034890

[B64] Pereda-Miranda R. Kaatz G. W. Gibbons S. (2006). Polyacylated oligosaccharides from medicinal Mexican morning glory species as antibacterials and inhibitors of multidrug resistance in *Staphylococcus aureus* . J. Nat. Prod. 69, 406–409. 10.1021/np050227d 16562846

[B65] Perumal S. Mahmud R. (2013). Chemical analysis, inhibition of biofilm formation and biofilm eradication potential of Euphorbia hirta L. against clinical isolates and standard strains. BMC Complement. Altern. Med. 13, 346. 10.1186/1472-6882-13-346 24321370 PMC4029191

[B66] Peterson L. R. (2008). A review of tigecycline-the first glycylcycline. Int. J. Antimicrob. Agents 32 (4), S215–S222. 10.1016/S0924-8579(09)70005-6 19134522

[B67] Ponnusamy K. Ramasamy M. Savarimuthu I. Paulraj M. G. (2010). Indirubin potentiates ciprofloxacin activity in the NorA efflux pump of *Staphylococcus aureus* . Scand. J. Infect. Dis. 42, 500–505. 10.3109/00365541003713630 20380543

[B68] Qian M. Tang S. Wu C. Wang Y. He T. Chen T. (2015). Synergy between baicalein and penicillins against penicillinase-producing *Staphylococcus aureus* . Int. J. Med. Microbiol. 305, 501–504. 10.1016/j.ijmm.2015.05.001 26028441

[B69] Qin R. Xiao K. Li B. Jiang W. Peng W. Zheng J. (2013). The combination of catechin and epicatechin callate from Fructus Crataegi potentiates beta-lactam antibiotics against methicillin-resistant staphylococcus aureus (MRSA) *in vitro* and *in vivo* . Int. J. Mol. Sci. 14, 1802–1821. 10.3390/ijms14011802 23325048 PMC3565349

[B70] Quiles-Melero I. Gómez-Gil R. Romero-Gómez M. P. Sánchez-Díaz A. M. de Pablos M. García-Rodriguez J. (2013). Mechanisms of Linezolid resistance among Staphylococci in a tertiary hospital. J. Clin. Microbiol. 51, 998–1001. 10.1128/JCM.01598-12 23269737 PMC3592090

[B71] Richter S. G. Elli D. Kim H. K. Hendrickx A. P. A. Sorg J. A. Schneewind O. (2013). Small molecule inhibitor of lipoteichoic acid synthesis is an antibiotic for Gram-positive bacteria. Proc. Natl. Acad. Sci. U. S. A. 110, 3531–3536. 10.1073/pnas.1217337110 23401520 PMC3587227

[B72] Roemer T. Schneider T. Pinho M. G. (2013). Auxiliary factors: A chink in the armor of MRSA resistance to β-lactam antibiotics. Curr. Opin. Microbiol. 16, 538–548. 10.1016/j.mib.2013.06.012 23895826

[B73] Rohrer S. Berger-Bächi B. (2003). FemABX peptidyl transferases: A link between branched-chain cell wall peptide formation and beta-lactam resistance in gram-positive cocci. Antimicrob. Agents Chemother. 47, 837–846. 10.1128/AAC.47.3.837-846.2003 12604510 PMC149326

[B74] Saravanakumar K. Chelliah R. Hu X. Oh D. H. Kathiresan K. Wang M. H. (2019). Antioxidant, anti-lung cancer, and anti-bacterial activities of Toxicodendron vernicifluum. Biomolecules 9, E127. 10.3390/biom9040127 PMC6523688 30934938

[B75] Scheffers D. J. Pinho M. G. (2005). Bacterial cell wall synthesis: New insights from localization studies. Microbiol. Mol. Biol. Rev. 69, 585–607. 10.1128/MMBR.69.4.585-607.2005 16339737 PMC1306805

[B76] Schmitz F. J. Petridou J. Fluit A. C. Hadding U. Peters G. von Eiff C. (2000). Distribution of macrolide-resistance genes in *Staphylococcus aureus* blood-culture isolates from fifteen German University hospitals. M.A.R.S. Study Group. Multicentre Study on Antibiotic Resistance in Staphylococci. Eur. J. Clin. Microbiol. Infect. Dis. 19, 385–387. 10.1007/s100960050500 10898143

[B77] Severin A. Wu S. W. Tabei K. Tomasz A. (2005). High-level (beta)-lactam resistance and cell wall synthesis catalyzed by the mecA homologue of Staphylococcus sciuri introduced into *Staphylococcus aureus* . J. Bacteriol. 187, 6651–6658. 10.1128/JB.187.19.6651-6658.2005 16166526 PMC1251583

[B78] Sewell E. W. C. Brown E. D. (2014). Taking aim at wall teichoic acid synthesis: New biology and new leads for antibiotics. J. Antibiot. (Tokyo) 67, 43–51. 10.1038/ja.2013.100 24169797

[B79] Shin J. Prabhakaran V. S. Kim K. S. (2018). The multi-faceted potential of plant-derived metabolites as antimicrobial agents against multidrug-resistant pathogens. Microb. Pathog. 116, 209–214. 10.1016/j.micpath.2018.01.043 29407230

[B80] Shiota S. Shimizu M. Mizushima T. Ito H. Hatano T. Yoshida T. (1999). Marked reduction in the minimum inhibitory concentration (MIC) of beta-lactams in methicillin-resistant *Staphylococcus aureus* produced by epicatechin gallate, an ingredient of green tea (Camellia sinensis). Biol. Pharm. Bull. 22, 1388–1390. 10.1248/bpb.22.1388 10746177

[B81] Shiota S. Shimizu M. Mizusima T. Ito H. Hatano T. Yoshida T. (2000). Restoration of effectiveness of beta-lactams on methicillin-resistant *Staphylococcus aureus* by tellimagrandin I from rose red. FEMS Microbiol. Lett. 185, 135–138. 10.1111/j.1574-6968.2000.tb09051.x 10754237

[B82] Shiota S. Shimizu M. Sugiyama J. Morita Y. Mizushima T. Tsuchiya T. (2004). Mechanisms of action of corilagin and tellimagrandin I that remarkably potentiate the activity of beta-lactams against methicillin-resistant *Staphylococcus aureus* . Microbiol. Immunol. 48, 67–73. 10.1111/j.1348-0421.2004.tb03489.x 14734860

[B83] Smith E. C. J. Kaatz G. W. Seo S. M. Wareham N. Williamson E. M. Gibbons S. (2007). The phenolic diterpene totarol inhibits multidrug efflux pump activity in *Staphylococcus aureus* . Antimicrob. Agents Chemother. 51, 4480–4483. 10.1128/AAC.00216-07 17664318 PMC2168009

[B84] Spížek J. Novotná J. Rezanka T. Demain A. L. (2010). Do we need new antibiotics? The search for new targets and new compounds. J. Ind. Microbiol. Biotechnol. 37, 1241–1248. 10.1007/s10295-010-0849-8 21086099

[B85] Stermitz F. R. Lorenz P. Tawara J. N. Zenewicz L. A. Lewis K. (2000a). Synergy in a medicinal plant: Antimicrobial action of berberine potentiated by 5’-methoxyhydnocarpin, a multidrug pump inhibitor. Proc. Natl. Acad. Sci. U. S. A. 97, 1433–1437. 10.1073/pnas.030540597 10677479 PMC26451

[B86] Stermitz F. R. Tawara-Matsuda J. Lorenz P. Mueller P. Zenewicz L. Lewis K. (2000b). 5’-Methoxyhydnocarpin-D and pheophorbide A: Berberis species components that potentiate berberine growth inhibition of resistant *Staphylococcus aureus* . J. Nat. Prod. 63, 1146–1149. 10.1021/np990639k 10978214

[B87] Swoboda J. G. Campbell J. Meredith T. C. Walker S. (2010). Wall teichoic acid function, biosynthesis, and inhibition. Chembiochem 11, 35–45. 10.1002/cbic.200900557 19899094 PMC2798926

[B88] Swoboda J. G. Meredith T. C. Campbell J. Brown S. Suzuki T. Bollenbach T. (2009). Discovery of a small molecule that blocks wall teichoic acid biosynthesis in *Staphylococcus aureus* . ACS Chem. Biol. 4, 875–883. 10.1021/cb900151k 19689117 PMC2787957

[B89] Therien A. G. Huber J. L. Wilson K. E. Beaulieu P. Caron A. Claveau D. (2012). Broadening the spectrum of β-lactam antibiotics through inhibition of signal peptidase type I. Antimicrob. Agents Chemother. 56, 4662–4670. 10.1128/AAC.00726-12 22710113 PMC3421906

[B90] Theuretzbacher U. (2011). Resistance drives antibacterial drug development. Curr. Opin. Pharmacol. 11, 433–438. 10.1016/j.coph.2011.07.008 21862408

[B91] Tooke C. L. Hinchliffe P. Bragginton E. C. Colenso C. K. Hirvonen V. H. A. Takebayashi Y. (2019). β-Lactamases and β-Lactamase inhibitors in the 21st century. J. Mol. Biol. 431, 3472–3500. 10.1016/j.jmb.2019.04.002 30959050 PMC6723624

[B92] Tyers M. Wright G. D. (2019). Drug combinations: A strategy to extend the life of antibiotics in the 21st century. Nat. Rev. Microbiol. 17, 141–155. 10.1038/s41579-018-0141-x 30683887

[B93] Unni S. Siddiqui T. J. Bidaisee S. (2021). Reduced susceptibility and resistance to vancomycin of *Staphylococcus aureus*: A review of global incidence patterns and related genetic mechanisms. Cureus 13, e18925. 10.7759/cureus.18925 34812309 PMC8603868

[B94] van Heijenoort J. (2007). Lipid intermediates in the biosynthesis of bacterial peptidoglycan. Microbiol. Mol. Biol. Rev. 71, 620–635. 10.1128/MMBR.00016-07 18063720 PMC2168651

[B95] van Heijenoort J. (2001). Recent advances in the formation of the bacterial peptidoglycan monomer unit. Nat. Prod. Rep. 18, 503–519. 10.1039/a804532a 11699883

[B96] Vestergaard M. Frees D. Ingmer H. (2019). Antibiotic resistance and the MRSA problem. Microbiol. Spectr. 7. 10.1128/microbiolspec.GPP3-0057-2018 PMC11590431 30900543

[B97] Vickery C. R. Wood B. M. Morris H. G. Losick R. Walker S. (2018). Reconstitution of *Staphylococcus aureus* lipoteichoic acid synthase activity identifies Congo Red as a selective inhibitor. J. Am. Chem. Soc. 140, 876–879. 10.1021/jacs.7b11704 29300473 PMC5856125

[B98] Wang D. Xie K. Zou D. Meng M. Xie M. (2018). Inhibitory effects of silybin on the efflux pump of methicillin-resistant *Staphylococcus aureus* . Mol. Med. Rep. 18, 827–833. 10.3892/mmr.2018.9021 29845191 PMC6059712

[B99] Wang S. Y. Sun Z. L. Liu T. Gibbons S. Zhang W. J. Qing M. (2014). Flavonoids from Sophora moorcroftiana and their synergistic antibacterial effects on MRSA. Phytother. Res. 28, 1071–1076. 10.1002/ptr.5098 24338874

[B100] Warren J. (2011). Drug discovery: Lessons from evolution. Br. J. Clin. Pharmacol. 71, 497–503. 10.1111/j.1365-2125.2010.03854.x 21395642 PMC3080636

[B101] Weidenmaier C. Kokai-Kun J. F. Kristian S. A. Chanturiya T. Kalbacher H. Gross M. (2004). Role of teichoic acids in *Staphylococcus aureus* nasal colonization, a major risk factor in nosocomial infections. Nat. Med. 10, 243–245. 10.1038/nm991 14758355

[B102] Wright G. D. (2016). Antibiotic adjuvants: Rescuing antibiotics from resistance. Trends Microbiol. 24, 862–871. 10.1016/j.tim.2016.06.009 27430191

[B103] Wüthrich D. Cuénod A. Hinic V. Morgenstern M. Khanna N. Egli A. (2019). Genomic characterization of inpatient evolution of MRSA resistant to daptomycin, vancomycin and ceftaroline. J. Antimicrob. Chemother. 74, 1452–1454. 10.1093/jac/dkz003 30726929

[B104] Xu Z. Li L. Shirtliff M. E. Peters B. M. Li B. Peng Y. (2011). Resistance class 1 integron in clinical methicillin-resistant *Staphylococcus aureus* strains in southern China, 2001-2006. Clin. Microbiol. Infect. 17, 714–718. 10.1111/j.1469-0691.2010.03379.x 21521411

[B105] Yahav D. Giske C. G. Grāmatniece A. Abodakpi H. Tam V. H. Leibovici L. (2020). New β-Lactam-β-Lactamase inhibitor combinations. Clin. Microbiol. Rev. 34, e00115–e00120. 10.1128/CMR.00115-20 PMC7667665 33177185

[B106] Zhang P. Miao X. Zhou L. Cui B. Zhang J. Xu X. (2020). Characterization of Oxacillin-susceptible mecA-positive *Staphylococcus aureus* from food poisoning outbreaks and retail foods in China. Foodborne Pathog. Dis. 17, 728–734. 10.1089/fpd.2019.2774 32716657

[B107] Zhao W. H. Hu Z. Q. Hara Y. Shimamura T. (2002). Inhibition of penicillinase by epigallocatechin gallate resulting in restoration of antibacterial activity of penicillin against penicillinase-producing *Staphylococcus aureus* . Antimicrob. Agents Chemother. 46, 2266–2268. 10.1128/AAC.46.7.2266-2268.2002 12069986 PMC127279

[B108] Zhao W. H. Hu Z. Q. Okubo S. Hara Y. Shimamura T. (2001). Mechanism of synergy between epigallocatechin gallate and beta-lactams against methicillin-resistant *Staphylococcus aureus* . Antimicrob. Agents Chemother. 45, 1737–1742. 10.1128/AAC.45.6.1737-1742.2001 11353619 PMC90539

[B109] Zhou X. Jia F. Liu X. Yang J. Zhang Y. Wang Y. (2011). *In vitro* synergistic interaction of 5-O-Methylglovanon and Ampicillin against Ampicillin resistant *Staphylococcus aureus* and Staphylococcus epidermidis isolates. Arch. Pharm. Res. 10, 1751–1757. 10.1007/s12272-011-1019-x 22076775

